# MDCrow: automating molecular dynamics workflows with large language models

**DOI:** 10.1088/2632-2153/ae4b07

**Published:** 2026-03-30

**Authors:** Quintina Campbell, Sam Cox, Jorge Medina, Brittany Watterson, Andrew D White

**Affiliations:** 1 Department of Chemical Engineering, University of Rochester, Rochester, NY, United States of America; 2 Department of Biomedical Engineering, University of Rochester, Rochester, NY, United States of America; 3 FutureHouse Inc., San Francisco, CA, United States of America

**Keywords:** agent, agentic AI, computational biology, molecular dynamics, large language models

## Abstract

Molecular dynamics (MD) simulations are essential for understanding biomolecular systems but remain challenging to automate. Recent advances in large language models (LLMs) have demonstrated success in automating complex scientific tasks using LLM-based agents. In this paper, we introduce MDCrow, an agentic LLM assistant capable of automating MD workflows for proteins. MDCrow uses chain-of-thought over 40 expert-designed tools for handling and processing files, setting up simulations, analyzing the simulation outputs, and retrieving relevant information from literature and databases. We assess MDCrow’s performance across 25 common tasks of varying complexity, and we evaluate the agent’s robustness to difficulty and prompt style. gpt-4o is able to complete increasingly complex tasks with low variance, followed closely by llama3-405b, a compelling open-source model. While prompt style does not influence the best models’ performance, it has significant effects on smaller models.

## 1. Introduction

Molecular dynamics (MD) is a common method to understand dynamic and complex systems in chemistry and biology. While MD is now routine, its integration into and impact on scientific workflows has increased dramatically over the past few decades [1–3]. There are two main reasons for this: First, MD provides valuable insights. Through simulations, scientists can study structural and dynamic phenomena, perturbations, and dynamic processes in their chemical systems. Second, innovations in hardware and expert-designed software packages have made MD much more accessible to both experienced and novice users [3].

For a given protein simulation, parameter selection is nontrivial: the user must provide the input structure (such as a Protein Data Bank (PDB) [4] file), select a force field (e.g. CHARMM [5], AMBER [6]), and specify parameters such as temperature, integrator, simulation length, and equilibration protocols. Simulations also generally require pre- and post-processing steps, along with various analyses. For instance, a user may need to clean or trim a PDB file, add a solvent, or analyze the protein’s structure. After simulation, they might examine protein conformation over time or assess its stability under different conditions. The choices for pre-processing, analysis, and simulation parameters are highly specific to any given use case and often require expert intuition. In practice, even with careful parameter selection, one often needs to run simulations, evaluate results, and adjust settings iteratively. Thus, automating this process is difficult but beneficial.

Several efforts have been made to automate MD workflows, including Admiral [7], CHAPERONg [8], FabSim [9], the Simulation Foundry [10], Gmx_qk [11], MolAr [12], ProFESSA [13], ProtoCaller [14], SimStack [15], with most targeting specific application domains, such as RadonPy for polymer’s simulations [16], or PyAutoFEP for proteins and small molecules for drug-screening [17]. Other approaches are constrained to a particular combination of simulation software and simulation (e.g. GROMACS and Free Energy Perturbation). Certainly, there has been significant community-driven improvement in automating and creating MD toolkits (EasyAmber [18], PACKMOL [19], MDAnalysis [20], MDTraj [21], OpenMM [22], GROMACS [23], LAMMPS [24]) and user-friendly interfaces and visualizations (MDANSE [25], QwikMD [26], MDWiZ [27], METAGUI [28], VMD [29], Gromita [30], PlayMolecule [31]). While these advances improve the capabilities and ease of use, the inherent variability of MD workflows still poses a great challenge for full automation.

Large language model (LLM) agent frameworks, such as Toolformer [32], MRKL [33], ReAct [34], Aviary [35], have gained popularity for their ability to automate technical tasks through scientific reasoning and tool usage. LLM agents even surpass domain-specialized LLMs (e.g. BioGPT [36], Med-PaLM [37]) when programmed for specialized roles [38]. These agents have demonstrated promising results in scientific tasks within a predefined toolspace like ChemCrow [39], Coscientist [40], and CACTUS [41], successfully automating complex workflows and novel design in chemical synthesis. Similarly, LLM-driven automation has been explored in material science and chemistry research [42], including catalyst design [43], inorganic synthesis [44], scientific literature review [45], and data aggregation [46]. LLM-driven automation is also explored in more sophisticated tasks that involve multi-step or multi-agent systems: context-aware experiment planning [47], evaluation of protocol planning [48], gene editing experiment design [49], reaction development framework [50], metal organic framework generation [51], the scientific discovery pipeline with a team of AI scientists [52]. Most similar to this work, ProtAgents [53] is a multi-agent modeling framework tackling protein-related design and analysis, and LLaMP [54] applies a retrieval-augmented generation-based ReAct agent to simulate inorganic materials by interfacing with literature databases, Wikipedia, and atomistic simulation tools. Despite these advances, no fully adaptive and autonomous systems exist for biochemical MD or protein simulations. See Ramos *et al* [55] for an informative review on the design, assessment, and applications of scientific agents.

**Figure 1.**
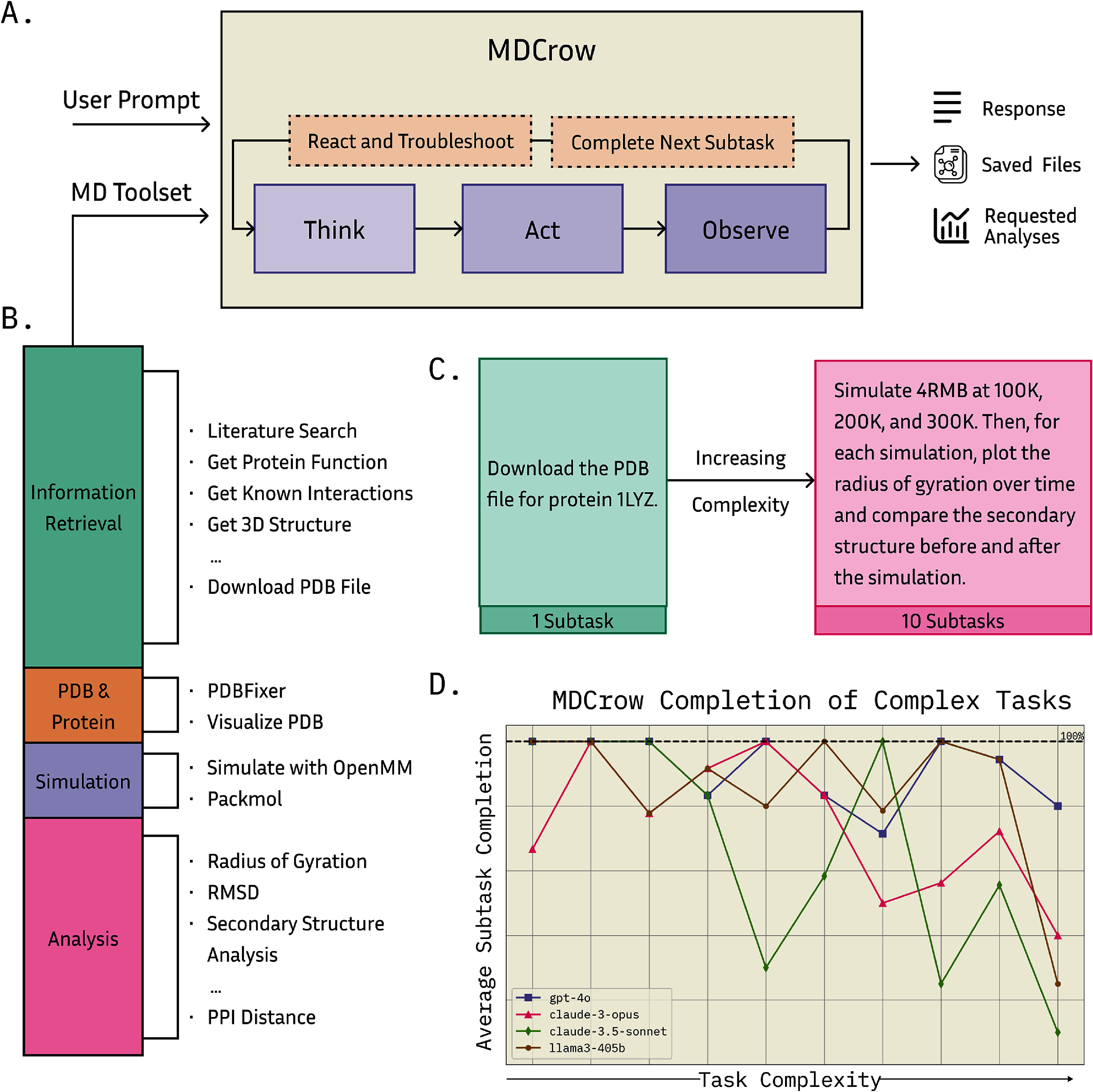
(A) MDCrow workflow. Starting with a user prompt and initialized with a set of MD tools, MDCrow follows a chain-of-thought process until it completes all tasks in the prompt. The final output includes a response, along with all resulting analyses and files. (B) The tool distribution categorized into four types: information retrieval, PDB and protein handling, simulation, and analysis. A few examples from each category are shown. (C) Two example prompts that MDCrow is tested on. The first is the simplest prompt, containing only 1 subtask. The most complex task requires 10 subtasks. (D) Average subtask completion across all 25 prompts as task complexity (the number of subtasks per prompt) increases. The top three performing base-LLMs are shown. Among them, gpt-4o and llama3-405b are consistently stable, staying close to 100% completion even as task complexity increases.

Here we present MDCrow, an LLM-agent capable of autonomously completing MD workflows (figure 1). We measure MDCrow’s performance across 25 common tasks with varying difficulty and compare performance of different LLM models. We then analyze the effect of prompting style on performance and assess performance against task complexity, based on required number of subtasks; finally, we compare MDCrow with an LLM equipped with a python interpreter with the required packages installed, rather than using a custom built environment. We find that MDCrow with gpt-4o or llama3-405b is able to perform nearly all of our assessed tasks and is relatively insensitive to how precise the instructions are given to it. See figure 1(D) for an overview of the main results.

Following this work, other recent LLM-based MD automation frameworks include NAMD-Agent [56], which prioritizes fast setup protein systems via web automation CHARMM-GUI, and DynaMate [57], which extends LLM automation to protein-ligand complexes through a multi-agent framework utilizing AmberTools and GROMACS. MDCrow focuses on protein simulations and uniquely contributes in the following ways: systematic benchmarking across open-source and frontier LLMs, assessing prompt-style robustness and capability of automating multi-step non-linear tasks of varying complexity, and checkpoint-based chat with memory for pausing and resuming across sessions.

## 2. Methods

### 2.1. MDCrow toolset

MDCrow consists of an environment with tools that emit observations and an LLM that selects appropriate actions (tools + input arguments). MDCrow is built with Langchain [58] and a ReAct style prompt [34]. The tools mostly consist of analysis and simulation methods; we use OpenMM [22] and MDTraj [21] packages, but in principle, our findings generalize to any such packages. See SI section **6** for an example of MDCrow adapting to work with GROMACS. The LLM agent operates at LLM temperature of 0.1, which is relatively low stochasticity and reflects realistic LLM agent behavior if one chooses to use LLMs to automate MD workflows.

MDCrow’s tools can be categorized in four groups: information retrieval, PDB & protein, simulation, and analysis (see figure 1(B)).

**Information retrieval tools** These tools enable MDCrow to build context and answer simple questions posed by the user. Most of the tools serve as wrappers for UniProt API functionalities [59], allowing access to data such as 3D structures, binding sites, and kinetic properties of proteins. Additionally, we include a LiteratureSearch tool, which uses PaperQA [45] to answer questions and retrieve information from literature. PaperQA accesses a local database of relevant PDFs, selected specifically for the test prompts, which can be found in SI section 3. This real-time information helps the system provide direct answers to user questions and can also assist the agent in selecting parameters or guiding simulation processes.

**PDB & protein tools** MDCrow uses these tools to interact directly with PDB files, performing tasks such as cleaning structures with PDBFixer [22], retrieving PDBs for small molecules and proteins, and generating visualizations of PDBs for the user through Molrender [60] or NGLview [61].

**Simulation tools** The simulation tools use OpenMM [22] and Packmol [19] for preprocessing and simulation. These tools are built to manage dynamic simulation parameters, handle errors related to inadequate parameters or incomplete preprocessing, and address missing forcefield templates efficiently. The agent responds to simulation setup errors through informative error messages, improving overall robustness. Finally, the simulation tools outputs Python scripts that can be modified directly by MDCrow whenever the simulation requires additional steps or parameters.

**Analysis tools** This group of tools is the largest in the toolset, designed to cover common MD analysis methods, many of which are built on MDTraj [21] functionalities. Examples include computing the root mean squared distance (RMSD) with respect to a reference structure, calculating the radius of gyration, analyzing the secondary structure, and generating plots.

### 2.2. Chatting

**Figure 2.**
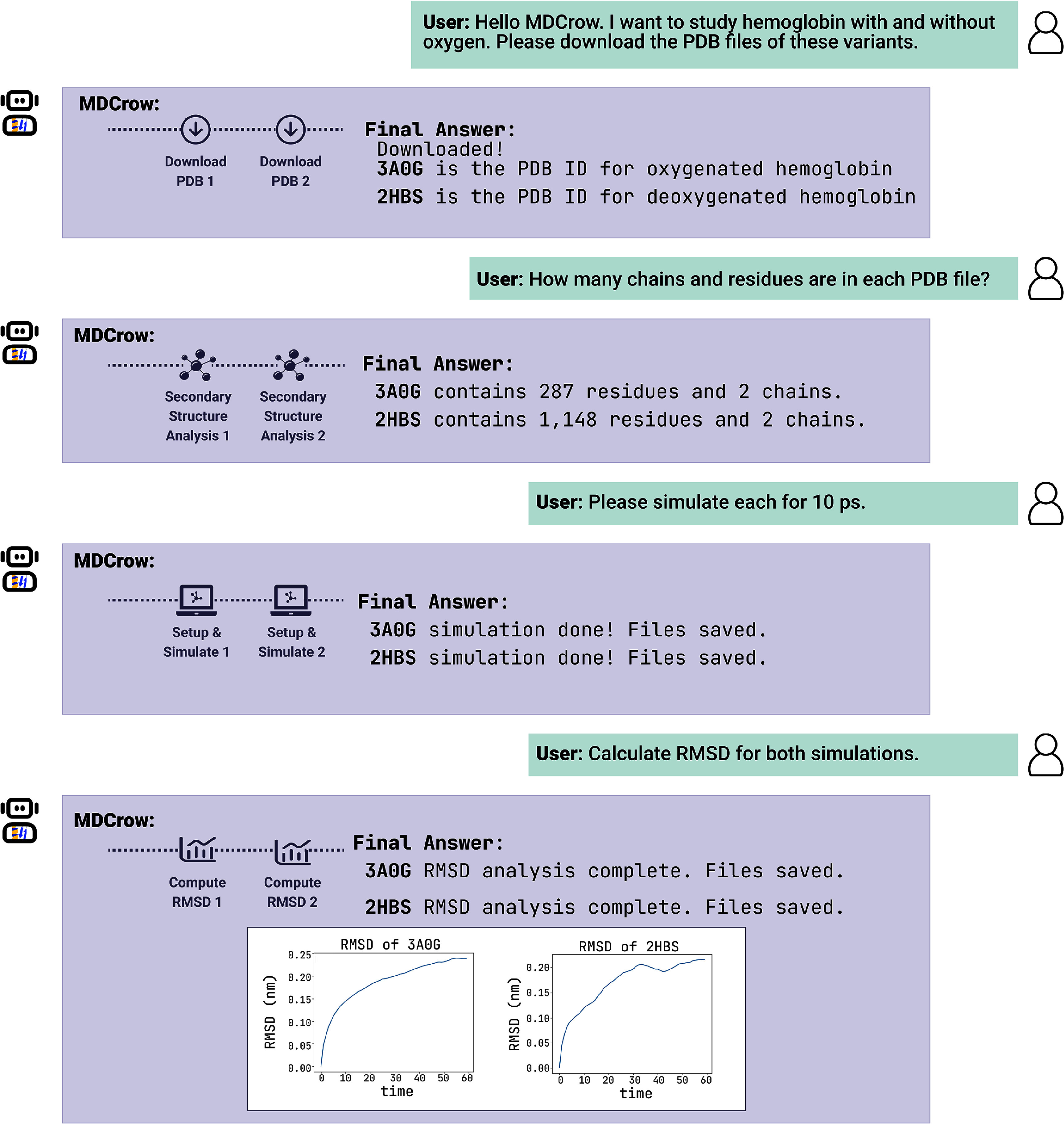
Example of chat with MDCrow. The user first asks to download PDB files for two systems. Then, once MDCrow has completed this task, the user asks for analysis of the files. Next, the user asks for a quick 10 ps simulation of both files, and MDCrow saves all files for later handling. Lastly, the user asks for plots of RMSD for each simulation over time, and MDCrow responds with each plot.

A key challenge in developing an automated MD assistant and supporting user interactions with simulations is ensuring it can manage a large number of files, analyses, and long simulations and runtimes. Although MDCrow has been primarily tested with shorter simulations, it is designed to handle larger workflows as well. Its ability to retrieve and resume previous runs allows users to start a simulation, step away during the long process, and later continue interactions and analyses without needing to stay engaged the entire time. When returning, users can easily build on prior sessions–for example, asking for additional plots or modified conditions (e.g. running again at a lower temperature). An example of this chatting feature is shown in figure 2.

MDCrow creates an LLM-generated summary of the user prompt and agent trace, which is assigned to a unique run identifier provided at the end of the run (but accessible at any time during the session). Each run’s files, figures, and path registry are saved in a unique checkpoint folder linked to the run identifier.

When resuming a chat, the LLM loads the summarized context of previous steps and maintains access to the same file corpus, as long as the created files remain accessible. To resume a run, the user simply provides the checkpoint directory and run identifier. MDCrow then loads the corresponding memory summaries and retrieves all associated files, enabling seamless continuation of analyses.

## 3. Results

### 3.1. MDCrow performance on various tasks

To assess MDCrow’s ability to complete tasks of varying difficulty, we designed 25 prompts with different levels of complexity and documented the number of subtasks (minimum required steps) needed to complete each task. MDCrow was not penalized for taking additional steps, but was penalized for omitting necessary ones. For example, the first prompt in figure 1(C) contains a single subtask, whereas the complex task requires 10 subtasks: downloading the PDB file, performing three simulations, and performing two analyses per simulation. If the agent failed to complete an earlier step, it was penalized for every subsequent step it could not perform due to that failure.

**Figure 3.**
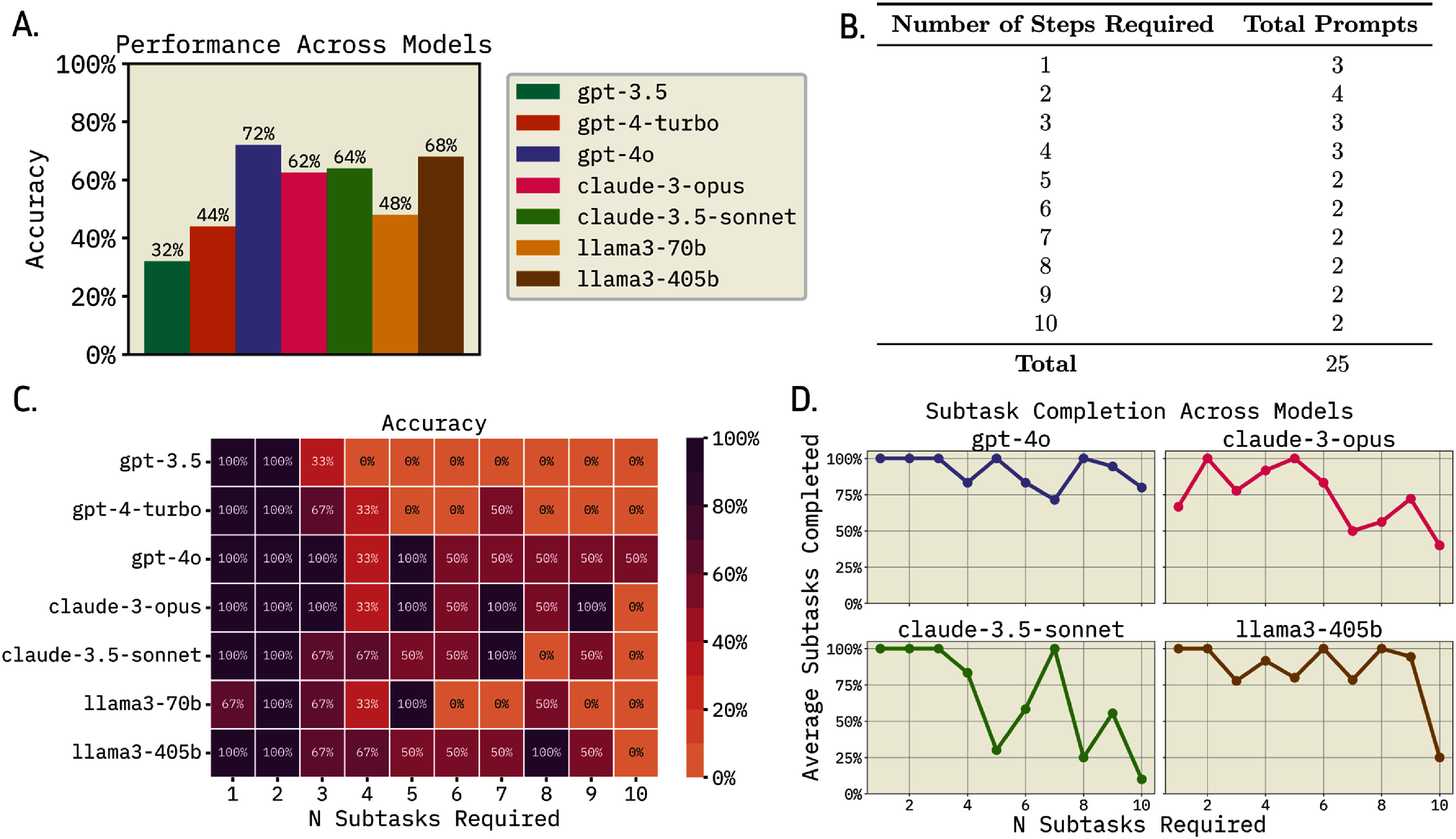
MDCrow performance across large language models. (A) Summary of MDCrow performance dependent on LLM. Percentage of accuracy is determined by whether it gave acceptable final answer or not. While statistically indistinguishable from Claude and Llama models, gpt-4o significantly outperforms the rest of GPT models on giving accurate solutions (*t*-test, 0.004 $\unicode{x2A7D}$ *p*-value $\unicode{x2A7D} 0.046$). (B) The distribution of number of subtasks in each task across 25 prompts. The prompts range from 1 to 10 steps, with each step count belonging to at least 2 prompts. (C) Percentages of prompts with accurate solutions with respect to LLM used and number of subtasks per task. The correlation between accuracy and complexity is statistically significant for all LLMs (Spearman correlation, $3.9\times10^{-7} \unicode{x2A7D}$ *p*-value $\unicode{x2A7D} 1.1\times10^{-2}$). (D) Percentage of the subtasks that the agent completed for each base LLM per task.

The 25 prompts require between 1 and 10 subtasks, with their distribution shown in figure 3(B). Each prompt was tested across three GPT models (gpt-3.5-turbo-0125, gpt-4-turbo-2024-04-09, gpt-4o-2024-08-06) [62, 63], two Llama models (llama-v3p1-405b-instruct, llama-v3p1-70b-instruct) [64] (accessed via the Fireworks AI API with 8-bit floating point (8FP) quantization [65]), and two Claude models (claude-3-opus-20 240 229, claude-3-5-sonnet-20 240 620) [66, 67]. A newer Claude Sonnet model, claude-3-5-sonnet-20 241 022 was tested in later experiments but was not found to give superior results, so it was not tested on these 25 prompts. All other parameters were held constant across tests, and each version of MDCrow executed a single run per prompt.

Each run was evaluated by experts recording the number of required subtasks the agent completed and using Boolean indicators to indicate accuracy, whether the agent triggered a runtime error, and whether the trajectory contained any hallucinations. Since the agent trajectories for each run are inherently variable, accuracy is defined as the result’s consistency with the expected trajectory rather than comparing against a fixed reference.

The percentage of tasks that were deemed to have valid solutions for MDCrow with each base-LLM is shown in figure 3(A). The lowest performing model was gpt-3.5. This is not surprising, as this model had some of the highest hallucination rates (32% of prompt completions contained hallucinations), compared to the absence of documented hallucinations in the higher performing models, gpt-4o and llama3-405b. However, the discrepancy in accuracy rates between models cannot solely be attributed to hallucinations, as gpt-3.5 attempted fewer than half of the required subtasks, whereas the higher-performing models, gpt-4o and llama3-405b, attempted 80%–90% of the required subtask, earning accuracy in answering for 72% and 68% of tasks, respectively (figures 3(C) and (D)).

These results indicate that MDCrow can handle complex MD tasks but is limited by the capabilities of the base model. For gpt-4-turbo, gpt-3.5, and llama3-70b, the number of trajectories with verified results decreases significantly as task complexity increases (figure 3(C)). In contrast, gpt-4o and llama3-405b show only a slight decline, demonstrating that MDCrow performs well even for complex tasks when paired with more robust base models.

### 3.2. MDCrow robustness

**Figure 4.**
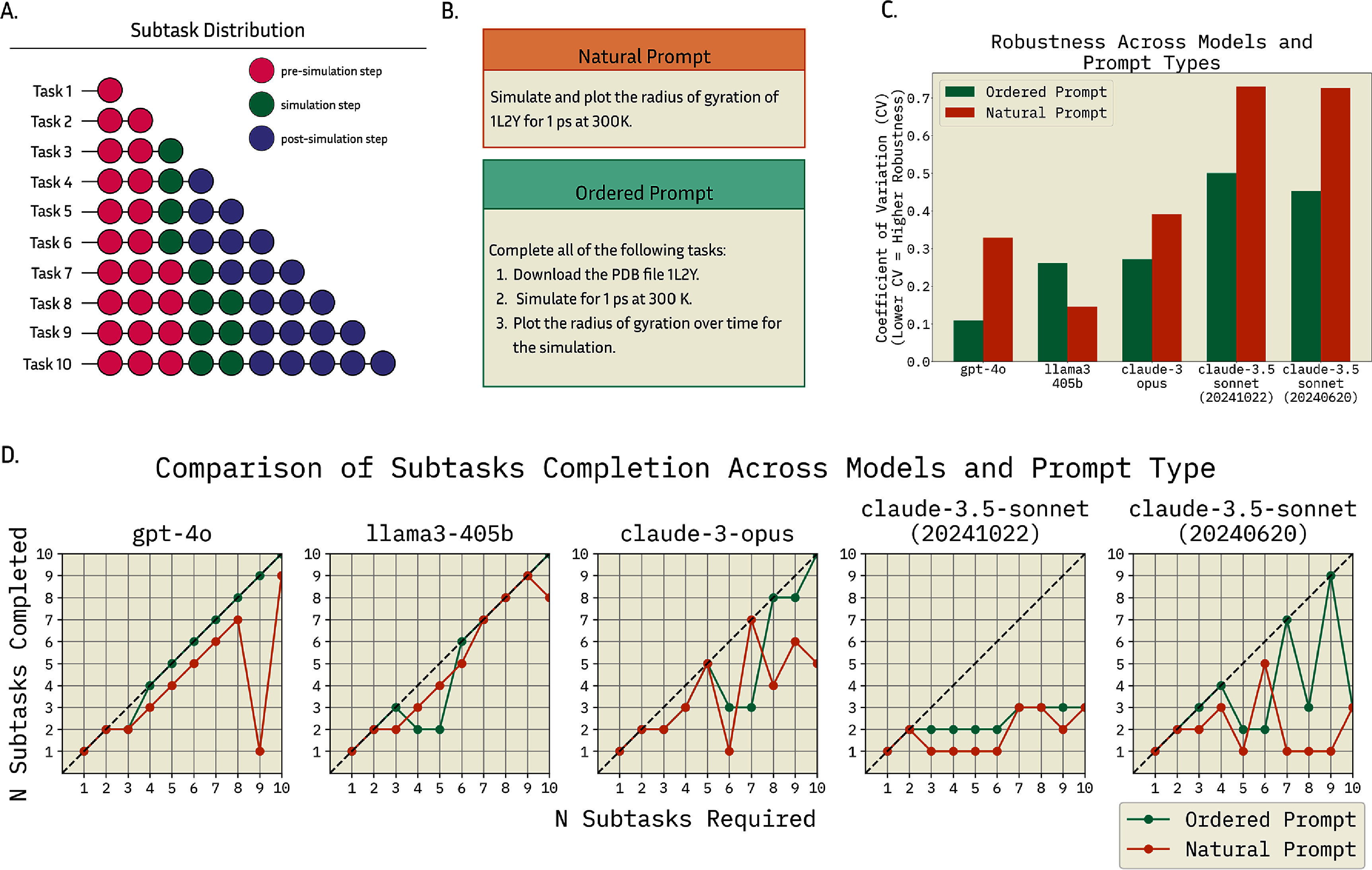
(A) The number of subtasks in each task, categorized by type. Task 1 begins with a single pre-simulation subtask (*Download a PDB file*) and each subsequent task adds a single subtask, adding to a total of 10 tasks with a maximum of 10 subtasks. (B) Example of ‘Natural’ and ‘Ordered’ prompt style on a three-step prompt. (C) The robustness of MDCrow built on each model with both prompt types, measured by coefficient of variation (CV). Lower CV is interpreted as greater consistency. gpt-4o and llama3-405b are the more robust models, as the Claude models have higher CVs. (D) Comparison of subtask completion across models and prompt types. In the 9-subtask prompt, gpt-4o encountered an error after only one step and gave up without trying to fix it. In general, gpt-4o and llama3-405b have relatively robust performance with increasing complexity for both prompt types. claude-3-opus struggles with more complex tasks, making more logical errors for complex tasks. The two claude-3.5-sonnet models showed fairly poor performance across this experiment.

We evaluated MDCrow’s robustness on complex prompts and different prompt styles. We hypothesized that some models would excel at completing complex tasks, while others would struggle–either forgetting steps or hallucinating–as the number of required subtasks increased. To test this, we created a sequence of 10 prompts that increased in complexity. The first prompt required a single subtask, and each subsequent prompt added an additional subtask (see figure 4(A)). Each prompt was tested twice: once in a natural, conversational style and once with explicitly ordered steps. Example prompts can be seen in figure 4(B).

To quantify robustness, we calculated the coefficient of variation (CV) for the percentage of completed subtasks across tasks. A lower CV indicates greater consistency in task completion and, therefore, higher robustness. The analysis revealed clear differences in robustness across models and prompt types. Overall, gpt-4o and llama3-405b demonstrated moderate to high robustness, while the Claude models showed significantly lower robustness. The performance comparison is shown in figure 4(C).

We expected that the percentage of subtasks completed by each model would decrease as task complexity increased. However, with gpt-4o and llama3-405b as base models, MDCrow demonstrated a strong relationship between the number of required and completed subtasks (figure 4(D)) for both prompt types, indicating consistent performance regardless of task complexity or prompt style. The three included Claude models demonstrated less impressive performance. claude-3-opus followed the linear trend very loosely, becoming more erratic as task complexity increased. As the tasks required more subtasks, the model consistently misses nuances in the instructions and makes logical errors. Both claude-3.5-sonnet models gave poor performance on these tasks, often producing the same error (see SI section 1).

### 3.3. MDCrow comparison

We also compared MDCrow to two baselines: a ReAct [34] agent with only a Python REPL tool and a zero-shot LLM. MDCrow and the baselines were tested on the same 25 prompts as previously mentioned, all using gpt-4o. We use different system prompts to accommodate each framework, guiding the LLM to utilize common packages with MDCrow, and these prompts can be found in SI section 2.

**Figure 5.**
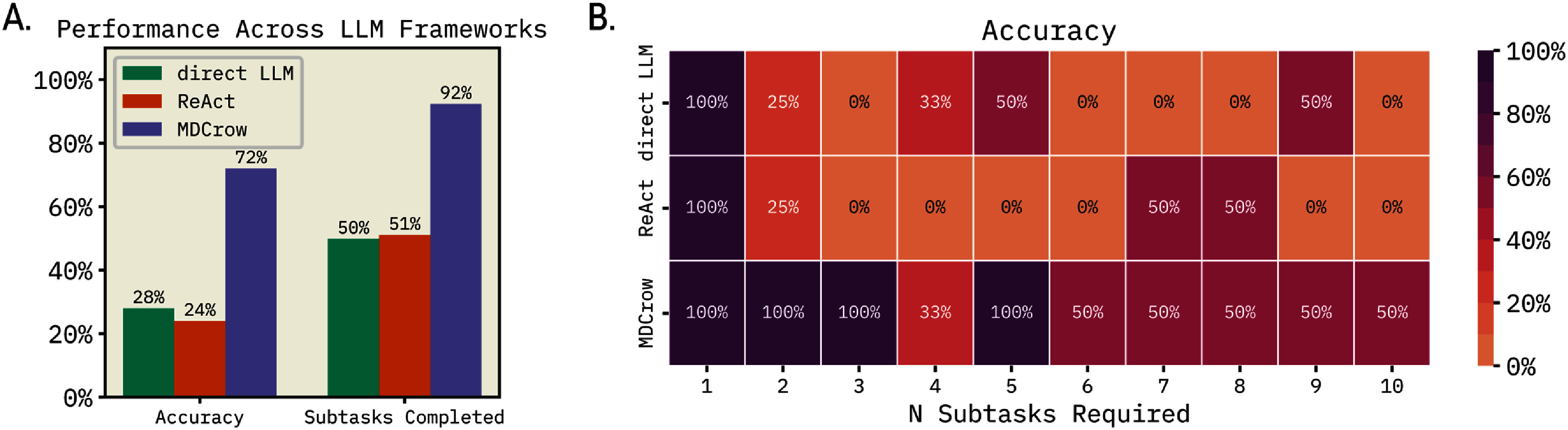
Performance across LLM Frameworks using the same 25-prompt set: MDCrow, direct LLM with no tools (single-query), and ReAct agent with only Python REPL tool. All use gpt-4o. (A) Performance among LLM frameworks measured by whether accuracy and average percentage of subtasks they complete for each of 25 task prompts. MDCrow is significantly better at giving accurate solutions than direct LLM (*t*-test, $p = 1\times10^{-3}$) and ReAct (*t*-test, $p = 4\times10^{-4}$). MDCrow completes significantly more subtasks on average compared to direct LLM (*t*-test, $p = 1\times10^{-6}$) and ReAct (*t*-test, $p = 6\times10^{-6}$). (B) Percentage of tasks completed with the respect to LLM framework used and the number of subtasks required for each task. The correlation between accuracy and number of subtasks required is statistically significant, $p = 1\times10^{-3}$ for direct LLM and $p = 1\times10^{-4}$ MDCrow. The p value for ReAct is $p = 7\times10^{-2}$.

The single-query LLM is asked to complete the prompt by writing the code for all subtasks, not unlike what standalone ChatGPT would be asked to do. We then execute the code ourselves and evaluate the outcomes accordingly. ReAct with Python REPL can write, execute, and react to code using a chain-of-thought framework. We find that MDCrow outperforms the two baselines significantly, as shown in figure 5(A), in both subtask completion and accuracy. Both baseline methods struggled with code syntax errors and incorrect handling of PDB files, highlighting how human guidance and integrated tools improve reliability. There is not a significant difference between the two baselines, indicating that the ability to react to failures did not significantly boost performance of these coding-systems.

In figure 5(B), we observe that the performance of all three methods generally declines as task complexity increases. In fact, both baseline methods drop to zero after just three steps, with performance fluctuating erratically at higher complexities. This reflects the critical importance of proper file processing and simulation setup for achieving optimal LLM performance in MD tasks. In contrast, MDCrow demonstrates greater robustness and reliability in handling complex tasks, with its pre-defined toolset allowing accurate file processing and simulation setup, as well as its ability to dynamically adjust to errors.

## 4. Discussion

Although LLMs’ scientific abilities are growing [68–70], they cannot yet independently complete MD workflows, even with a ReAct framework and Python interpreter. However, with frontier LLMs, chain-of-thought, and an expert-curated toolset, MDCrow successfully handles a broad range of MD tasks. It performs 80% better than gpt-4o in ReAct workflows at completing subtasks, which is expected given the technical demands of MD workflows, such as managing complex files and recovering from simulation errors.

In some cases, particularly for complex tasks beyond its explicit toolset, MDCrow’s performance may improve with human guidance. The system’s chatting feature allows users to continue previous conversations, clarify misunderstandings, and guide MDCrow step-by-step through difficult tasks. This adaptability helps MDCrow recover from failures, refine its approach based on user intent, and handle more complex workflows. Thus, with more advanced LLM models, targeted feedback, and the addition of specialized tools, MDCrow could tackle an even broader range of tasks. Examples include annealing simulations, specifying appropriate box sizes, and using GROMACS instead of OpenMM, all of which can be found in SI. We did not do a full evaluation of MDCrow’s capabilities through this chatting feature in this work.

For all LLMs, task accuracy and subtask completion are affected by task complexity. Interestingly, while gpt-4o can handle multiple steps with the lowest variance, llama3-405b is a compelling second best, as an open-source model. Other models, such as gpt-3.5 and claude-3.5-sonnet, struggle with hallucinations or inability to follow multistep instructions. Performance on these models, however, is improved with explicit prompting or model-specific optimization (especially for claude-3.5-sonnet).

These tasks were focused on routine applications of MD with short simulation runtimes, limited to proteins, common solvents, and force fields included in the OpenMM package. We did not explore small-molecule force fields, especially related to ligand binding. We restricted this study to relatively straight-forward packages and examples, and in the future, MDCrow could be expanded to address more niche applications. Future work could also explore multi-modal approaches [71, 72] for tasks like convergence analysis or plot interpretations. The current framework relies on human-created tools and evaluation, but as LLM-agent systems become more autonomous [73], careful evaluation and benchmarking will be essential.

## 5. Conclusion

Running and analyzing MD simulations is non-trivial and hard to automate. Here, we explored using LLM agents to accomplish this. We built MDCrow, an LLM-agent and an environment consisting of over 40 common tools built for MD simulation and analysis. We found MDCrow could complete 72% of the tasks with the optimal settings (gpt-4o). llama-405B was able to complete 68%, providing a compelling open-source model. The best models were relatively robust to how the instructions are given, although weaker models struggle with unstructured instructions. Simply using an LLM with a python interpreter and required packages installed had a 28% accuracy. The performance of MDCrow was relatively stable as well, though dependent on the model. Correct assessment of these complex scientific workflows is challenging, and had to be done by hand. Chatting with the simulations, via extended conversations, is even more compelling, but is harder to assess.

Overall, this work demonstrates that LLM agents can meaningfully automate components of MD workflows. With careful setup, open-source models perform comparably to proprietary ones, indicating that capable scientific automation is possible with accessible models. As LLM capabilities advance and benchmarks are developed, systems like MDCrow could evolve to even more complex and autonomous tasks.

## Data Availability

The data that support the findings of this study are openly available at the following URL/DOI: https://doi.org/10.5281/zenodo.18193478 [74]. Supplementary data 1 available at https://doi.org/10.1088/2632-2153/ae4b07/data1.
